# Threshold magnetic field as a universal criterion for the selective transport of magnetized particles in microdroplets

**DOI:** 10.1038/s41598-023-36516-3

**Published:** 2023-06-09

**Authors:** Shinji Bono, Satoshi Konishi

**Affiliations:** 1 Research Organization of Science and Technology, Ritsumeikan University, Kusatsu, 525-8577 Japan; 2 Ritsumeikan Advanced Research Academy, Kyoto, 604-8502 Japan; 3 Ritsumeikan Global Innovation Research Organization, Ritsumeikan University, Kusatsu, 525-8577 Japan; 4 Department of Mechanical Engineering, College of Science and Engineering, Ritsumeikan University, Kusatsu, 525-8577 Japan

**Keywords:** Fluidics, Electrical and electronic engineering, Wetting

## Abstract

Transportation of magnetized particles (MPs) against gravity is possible by applying a magnetic field to the particles. This transport phenomenon of MPs in microdroplets can be quantitatively assessed by determining the contribution of individual forces acting on the MPs. We studied the selective transportation of MPs in microdroplets. MPs in microdroplets were transported in the opposite direction to gravity when we applied an external magnetic field larger than a threshold value. We modulated the intensity of the external magnetic field and selectively manipulated the MPs. As a result, MPs were separated into different microdroplets based on their magnetic properties. Our quantitative investigation of transport dynamics shows that the threshold magnetic field depends only on the magnetic susceptibility and the density of MPs. This is a universal criterion for the selective transport of magnetized targets such as magnetized cells in microdroplets.

## Introduction

Microdroplets, the typical volume of which is several microliters, can be regarded as discrete experimental systems for cell culture, such as micro-incubators, or micro-tubes. Three-dimensional digital microfluidics using microdroplets is a promising alternative to realize high-throughput biochemical assay^1–3^. The wetting pattern (WP) technique has been recently used for the manipulation of the shape of microdroplets^4,5^. When we introduce a water onto a substrate patterned with hydrophilic and hydrophobic materials, microdroplets are spontaneously formed. The shape of these microdroplets can be influenced by the relationship between the hydrophilic area and the quantity of water. Previous studies have reported that hydrophilic areas are regularly arranged, and the studies were successful in hanging a microdroplet array just below a WP substrate^2,6^. For example, in a hanging-droplet array, we can simultaneously culture cells in individual microdroplets under different conditions^7,8^.

The WP technique is useful not only for the formation of microdroplet arrays but also for their vertical contact control (VCC). In this process, the microdroplets on one end contact their opposite microdroplets, thus causing material transport^5^. For example, we can transport solute or particles from the top to the bottom microdroplet through gravity^6,9^. We can access several microdroplets in arrays simultaneously with a VCC process by applying the VCC to the droplet-array sandwiching technique (DAST)^10^. Moreover, we can perform selective VCC of arbitrary microdroplets in an array on a WP substrate integrated into the electro-wetting mechanism^9,11,12^. An application in biochemistry is where the VCC between the bottom and top microdroplets, which include cells, and histamines, respectively, triggers calcium oscillation reaction in the cells^10^. Therefore, the combination of VCC, and DAST serves as a high-throughput dispenser mechanism by replacing manual processes such as pipetting.

The transport of targets in microdroplets in the direction opposite to gravity develops three-dimensional target manipulation. The application of the external field is essential for transportation against gravity. Recently, a magnetic label of cells has been established and thus, the usage of magnetized cells has increased^13,14^. It may be possible to transport MPs against gravity by applying a magnetic field above the magnetized particles (MPs) such as magnetized cells. Application of manipulation using a magnetic field to microdroplet systems should contribute to the transport of targets in microdroplets in the direction opposite to gravity. Previous works reported magnetic levitation using diamagnetic interaction or the Meissner effect^15–17^. Considering the application to the manipulation of magnetized cells in water microdroplets, we focus on magnetic transport using the difference in magnetic susceptibility. The combination of magnetic transport of MPs and DAST may contribute to high-throughput biochemical assay, that is, the manipulation of magnetized cells cultured in microdroplets or magnetic chemical compounds synthesized in microdroplets.

The force applied by the magnetic field to the MPs depends on the magnetic properties of MPs^18^. The magnetic transport mechanism of MPs may serve as their separation mechanism based on their magnetic properties. MPs possessing high magnetic properties can be selectively transported by providing an appropriate unit of the external magnetic field. A combination of selective transport and VCC enables us to dispense MPs to different microdroplets. Magnetic transport may serve as a separation mechanism to dispense magnetized cells or magnetic chemical compounds to individual microdroplets based on their magnetic properties.

The integration of an external magnetic field application mechanism to DAST realizes the magnetic transport of MPs against gravity in microdroplets. Thus, for reliable transport, and separation, it is necessary to reveal factors that affect the selective transport of MPs. A magnetic field applied from the above drives force the MPs to migrate against gravity. MPs are transported upward only if the magnetic force is larger than the other resistance forces. MPs transported through microdroplets are subjected to gravity, buoyancy, and viscous forces. The attractive force driven by an external magnetic field should be proportional to the magnetic susceptibility of MPs. In contrast, the effect of gravity should be proportional to the density of MPs. To investigate the transport phenomenon of MPs in microdroplets quantitatively, the contribution of individual forces must be estimated quantitatively.

In this study, we selectively transported MPs in microdroplets against gravity and quantitatively investigated the effect of the magnetic properties of MPs on transport dynamics. To control the magnetic susceptibility and the density independently of each other, we fabricated model MPs. Further, we quantitatively investigated the relationship between the physical properties of MPs and their transport dynamics. In particular, we compared the theoretical prediction and magnetic transport behavior in the experiment and proposed the universal criterion for the selective transport of MPs in microdroplets.

## Results

**Figure 1:**
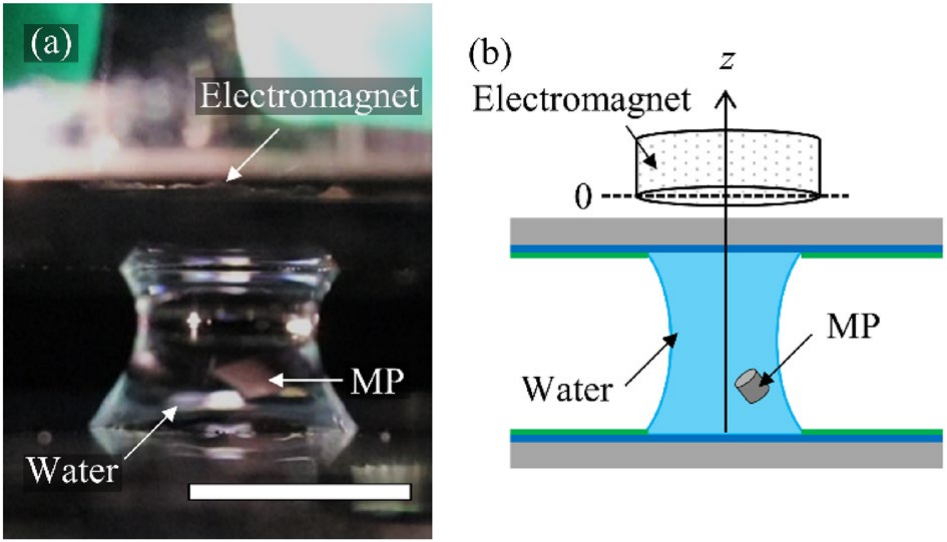
Experimental system of magnetic transport of a magnetized particle (MP) in a microdroplet. (**a**) Photograph of an MP in a coalescent microdroplet. Scale bar indicates 5 mm. (**b**) Schematics of our experimental system. We define the vertical direction as the *z*-axis.

Figure 1a shows a photograph of the experimental system used for this study. We formed 30-μL microdroplets on two WP substrates. After introducing an MP into a bottom microdroplet, top, and bottom WP substrates were fixed on *z*-axis control stages, so the two microdroplets were positioned opposite to each other. An electromagnet (FSGP-20, FUJITA) was set on the top WP substrate. In the experimental system, we maintained the distance between an MP and electromagnet *D* within 3.0–5.0 mm. We raised the bottom WP substrate to contact the top and bottom microdroplets to obtain a coalescent microdroplet. We applied current to the electromagnet connected with the direct current power supply (PMM25-1TA, KIKUSUI) and generated an external magnetic field from above. The magnetic field was directly proportional to the applied current. We separated a coalescent microdroplet into two while applying a magnetic field by lowering the bottom WP substrate.

Figure 1b shows the schematics of the experimental setup, where we defined the vertical direction as the *z*-axis. MP was initially located at *z* = −*D*. We also defined the magnetic force on a unit volume MP with χ = 1 as.

1 $${\text{f}}_{{\text{B}}} { = }\frac{{1}}{{\upmu _{{0}} }}{\text{B }}\frac{{\partial {\text{B}}}}{{\partial {\text{z}}}},$$

which is a control parameter to estimate the effect of the magnetic field. In the calibration experiments, we measured *f*_B_ using a Tesla meter (TM-601, KANETEC, Supplementary information 1).

**Figure 2:**
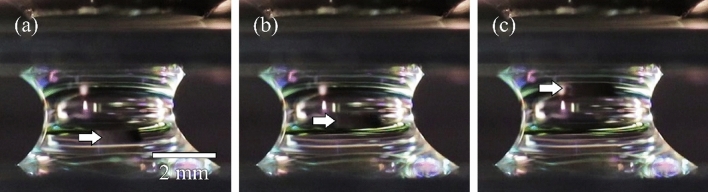
Magnetic transport of a magnetized particle (MP) in a microdroplet. (**a**) Photograph of the MP introduced in a coalescent microdroplet. ρ_MP_ and χ are 1.23 g cm^−3^ and 2.7 × 10^−2^, respectively. The MP in the coalescent microdroplet (**b**) 100 ms and (**c**) 200 ms after *f*_*B*_ = 7.7 μN mm^−3^ application. White arrows indicate the positions of the MP.

Figure 2a shows an MP introduced in a coalescent. ρ_MP_ and χ are 1.23 g cm^−3^ and χ  = 2.7 × 10^−2^, respectively. The MP was completely confined in the coalescent microdroplet. When we applied *f*_*B*_ = 7.7 μN mm^−3^ to the MP, the MP was transported upward (Fig. 2b: Supplementary movie 1). The MP contacted the top WP substrate and stopped transporting ~ 200 ms after *f*_*B*_ application (Fig. 2c). This result indicates that MPs in microdroplets can be transported against gravity by the external magnetic field. When we lowered the bottom WP substrate with *f*_*B*_, the MP was captured in a top microdroplet. We succeeded in transporting MPs from the bottom to the top microdroplets.

Next, we quantitatively investigated the transport velocity, *v*. We maintained ρ_MP_ as constant (1.23 g cm^−3^) and introduced MPs, whose χ s were different from each (Sample series 1), into microdroplets. We observed the position of MPs in microdroplets after applying *f*_*B*_ and obtain *v*.

**Figure 3:**
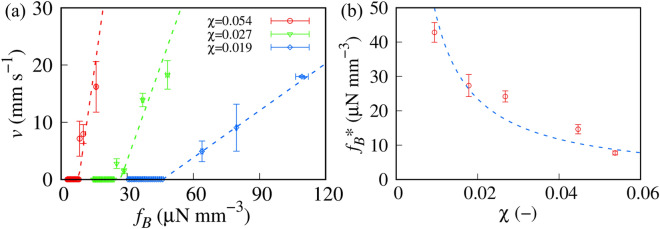
Transport behavior of magnetized particles (MPs) with constant ρ_MP_. (**a**) Transport velocity of MP, *v* as a function of magnetic force *f*_*B*_. ρ_MP_ is 1.23 g cm^−3^. The dashed lines are the best fits obtained using Eq. (2). (**b**) χ dependence of the threshold value *f*_B_*. The dashed line is the best fit obtained using an inversely proportional function of χ (*f*_B_* = β_1_/ χ, where β_1_ = 0.47 μN mm^−3^).

Figure 3a shows the *f*_*B*_ dependence of *v*. We confirmed that the gap of WP substrates in our experimental system was small enough for *v* to be regarded as constant. If the gap is wide, *f*_*B*_ cannot be regarded as constant. Thus, *v* should depend on the position with a wider gap. We did not observe the upward transport of MP with χ  = 0.054 (*v* = 0) if *f*_*B*_ was less than a threshold value *f*_B_* =  ~ 8 μN mm^−3^ (*f*_*B*_ < *f*_B_*). In contrast, for *f*_*B*_ > *f*_B_*, MP was transported upward with constant *v* which was found to be proportional to *f*_*B*_ (> *f*_B_*).*f*_B_* increases with decreasing χ, which suggests that a decrease in the magnetic susceptibility of MPs requires a large magnetic force. Besides, for *f*_*B*_ > *f*_B_*, the proportional constant of *v* to *f*_*B*_ (α = ∂*v*/∂*f*_*B*_) decreases with decreasing χ. To obtain *f*_B_* and α, we fit the experimental results using the following function,

2 $$v = \left\{ {\begin{array}{*{20}c} 0 \\ {\alpha (f_{B} - f_{B} *)} \\ \end{array} } \right. \begin{array}{*{20}c} {f_{B} < f_{B} *} \\ {f_{B} > f_{B} *} \\ \end{array}.$$

We superimposed the fitting results in Fig. 3a. We found that a linear function possessing a threshold value as Eq. (2) agrees with experimental results.

Figure 3b shows the χ dependence of *f*_B_*. The threshold value of magnetic transport *f*_B_* is inversely proportional to χ. Here, the dashed line in Fig. 3b is the best fit obtained using an inversely proportional function. χ can be used as a control parameter of *f*_B_*.

**Figure 4:**
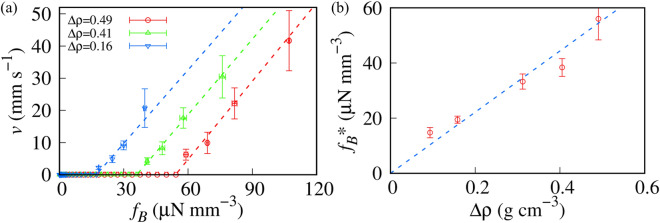
Transport behavior of magnetized particles (MPs) with constant χ (= 0.016). (**a**) Transport velocity of MP *v* as a function of magnetic force *f*_*B*_. The dashed lines are the best fits obtained using Eq. (2). (**b**) △ρ dependence of *f*_B_*. The dashed line is the best fit obtained using the linear function of △ρ (*f*_B_* =  β_2_ △ρ, where β_2_ = 0.11 N g^−1^).

To investigate the effect of the density, we maintained χ as constant (0.016) and introduced MPs, whose ρ_PM_s were different from each (Sample series 2). Figure 4a shows the *f*_*B*_ dependence of *v*. We defined the density difference with water (△ρ) as ρ_MP_−ρ_w_, where ρ_w_ = 0.997 g cm^−3^ is the density of water. Our observation was performed under the condition of △ρ > 0. We superimposed the best fits obtained using Eq. (2) as dashed lines in Fig. 4a. *f*_B_* monotonically increases with ρ. In contrast to χ, α  = ∂*v*/∂*f*_*B*_ is independent of △ρ.

Figure 4**b** shows the △ρ dependence of *f*_B_*. *f*_B_* is proportional to △ρ. The dashed line in Fig. 4b is the best fit obtained using the linear function of △ρ. Since MPs with △ρ < 0 are transported upward by buoyancy even without a magnetic field, *f*_B_* equals 0 with △ρ = 0. △ρ can also be used as a control parameter for *f*_B_*.

We found that *f*_B_* of magnetic transport can be controlled by both χ and △ρ. Further, we separated MPs based on the selective transport. First, we fabricated a high (high magnetic particle (HMP), χ  = 0.053) and a low magnetic particle (LMP, χ  = 0.018). We also fabricated a control particle (CP) that did not include Fe_3_O_4_, that is, χ  = 0. △ρs of HMP, LMP, and CP were 0.23 g cm^−3^. To distinguish between the particles based on their appearance, we set the diameters of cylindrical CP, LMP, and high magnetic particle (HMP) to 1.0, 1.5, and 2.0 mm, respectively. We summarized the physical properties of the target particles in Table 1.

**Table 1:** Physical properties of target particles for the selective transport.

Particle name	χ (−)	△ρ (g cm^−3^)	ϕ (mm)
CP	0	0.23	1.0
LMP	0.019	0.23	1.5
HMP	0.053	0.23	2.0

*CP* control particle; *LMP* low magnetic particle; *HMP* high magnetic particle.

**Figure 5:**
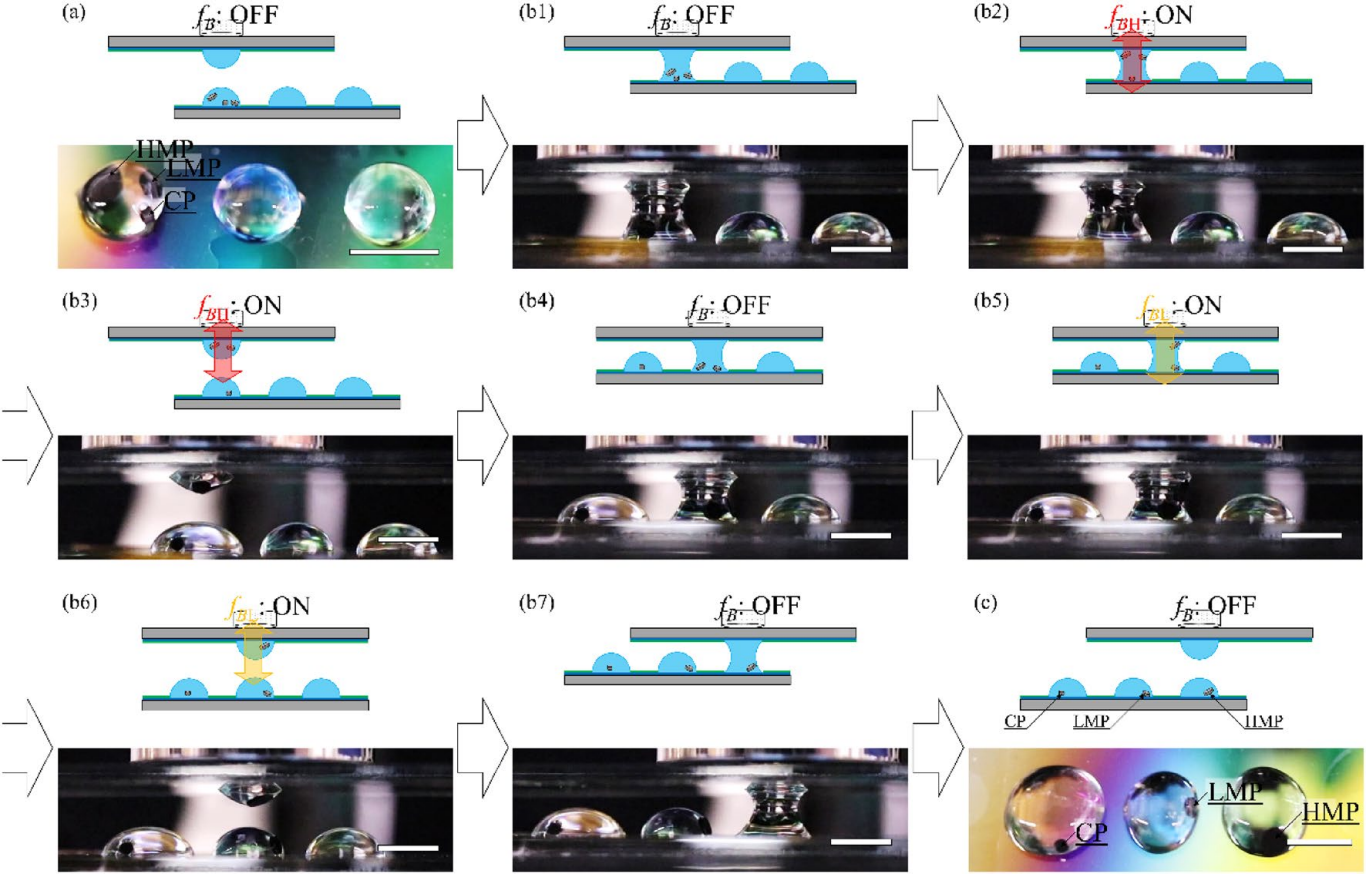
Selective transport of magnetized particles (MPs). (**a**) Top view picture of the initial state. We introduced control particle (CP), low magnetic particle (LMP), and high magnetic particle (HMP) into the left microdroplet. (**b1**) Top microdroplet contacts with the left microdroplet. (**b2**) We apply high magnetic force *f*_*B*H_ = 91 μN mm^−3^, and transport HMP, and LMP upward. (**b3**) After separating a coalescent microdroplet with *f*_*B*H_, HMP, and LMP are captured in the top microdroplet. (**b4**) Vertical contact control (VCC) between the top and the center microdroplets without a magnetic field causes downward transport of HMP and LMP. (**b5**) HMP is transported upward with low magnetic force *f*_*B*L_ = 13 μN mm^−3^. (**b6**) Separating the coalescent microdroplet with *f*_*B*L_, we capture HMP in the top microdroplets. (**b7**) After removing the magnetic field, we transport HMP downward through the VCC process. (**c**) Top view picture of microdroplet array after the selective transport. We dispense MPs to individual microdroplets by their magnetic susceptibility. Scale bars indicate 5 mm.

Figure 5a shows the initial condition of the selective transport. We introduced CP, LMP, and HMP into the left microdroplet. We positioned the top microdroplet so that the left microdroplet was opposite to the top microdroplet. Then, we performed the selective transport as follows (Supplementary movie 2): (b1). We raised the bottom WP substrate and performed VCC. (b2) After applying a high magnetic force *f*_*B*H_ = 91 μN mm^−3^ to the coalescent microdroplet, HMP, and LMP were transported upward while CP remained on the bottom WP substrate. (b3) We lowered the bottom WP substrate with *f*_*B*H_ and separated the coalescent microdroplet into two. The top microdroplet included HMP and LMP, while the bottom one included CP. (b4) After VCC between the top and the center microdroplets without magnetic field, HMP, and LMP were transported downward by gravity. (b5) Application of low magnetic force *f*_*B*L_ = 13 μN mm^−3^ caused the upward transport of HMP. (b6) Separation of the coalescent microdroplet with *f*_*B*L_ resulted in the separation of HMP and LMP into the top and bottom microdroplets, respectively. (b7) VCC between the top and the right microdroplets without a magnetic field caused the downward transport of HMP. (c) We observed microdroplets on the bottom WP substrate after selective transport and confirmed that CP, LMP, and HMP were included in the left, center, and right microdroplets, respectively.

Here, we used particles of different sizes. We also confirmed that the behavior of selective transport was independent of particle size. These results suggest that MPs in microdroplets were selectively transported by an external magnetic field depending on χ. As a result, MPs were dispensed to individual microdroplets. We also confirmed that MPs are selectively transported by an external magnetic field depending on △χ.

## Discussion

The dynamics of MPs in microdroplets under a magnetic field starting from the general equation of motion^18^ are presented as:

3 $$m\frac{dv}{{dt}} = F_{B} + F_{g} + F_{vis} ,$$

where *F*_*B*_, *F*_*g*_, and *F*_vis_ are the total magnetic field, gravity including buoyancy, and viscous force, respectively. From our observations, the *v* of MPs was constant. Thus, we neglected the inertia term on the left-hand side of Eq. (3).

The magnetic susceptibility and the density in the fabricated MPs were uniform. In our experiments, both sizes of MPs and the gap in the WP substrates were sufficiently small for *v* to be regarded as constant. Thus, we assume that *f*_*B*_ depends only on the applied current and is independent of the distance between the electromagnet and MPs. Based on the assumption, *F*_*B*_, and *F*_*g*_ on MPs are given as.

4.a $$F_{B} = \chi \chi_{{{\text{Fe}}_{{3}} {\text{O}}_{{4}} }} f_{B} V.$$

4.b $$F_{g} { = }{ - }\Delta \rho Vg,$$

where *V*, *g*, and χ_Fe3O4_ are the volume of an MP, gravitational acceleration, and magnetic susceptibility of bulk Fe_3_O_4_, respectively. For transporting MPs with *v*, *F*_vis_ is given as

4.c $$F_{{{\text{vis}}}} = - \xi v,$$

where ξ is the friction constant. For example, in the Stokes approximation for spherical microparticles with radius *r*, ξ = 6πη*r*, where η is viscous constant^19,20^.

Substituting Eq. (4) into Eq. (3), the transport velocity of MPs is given as

5 $$v = \frac{V}{\xi }\left( {\chi \chi_{{Fe_{3} O_{4} }} f_{B} - \Delta \rho g} \right).$$

**Figure 6:**
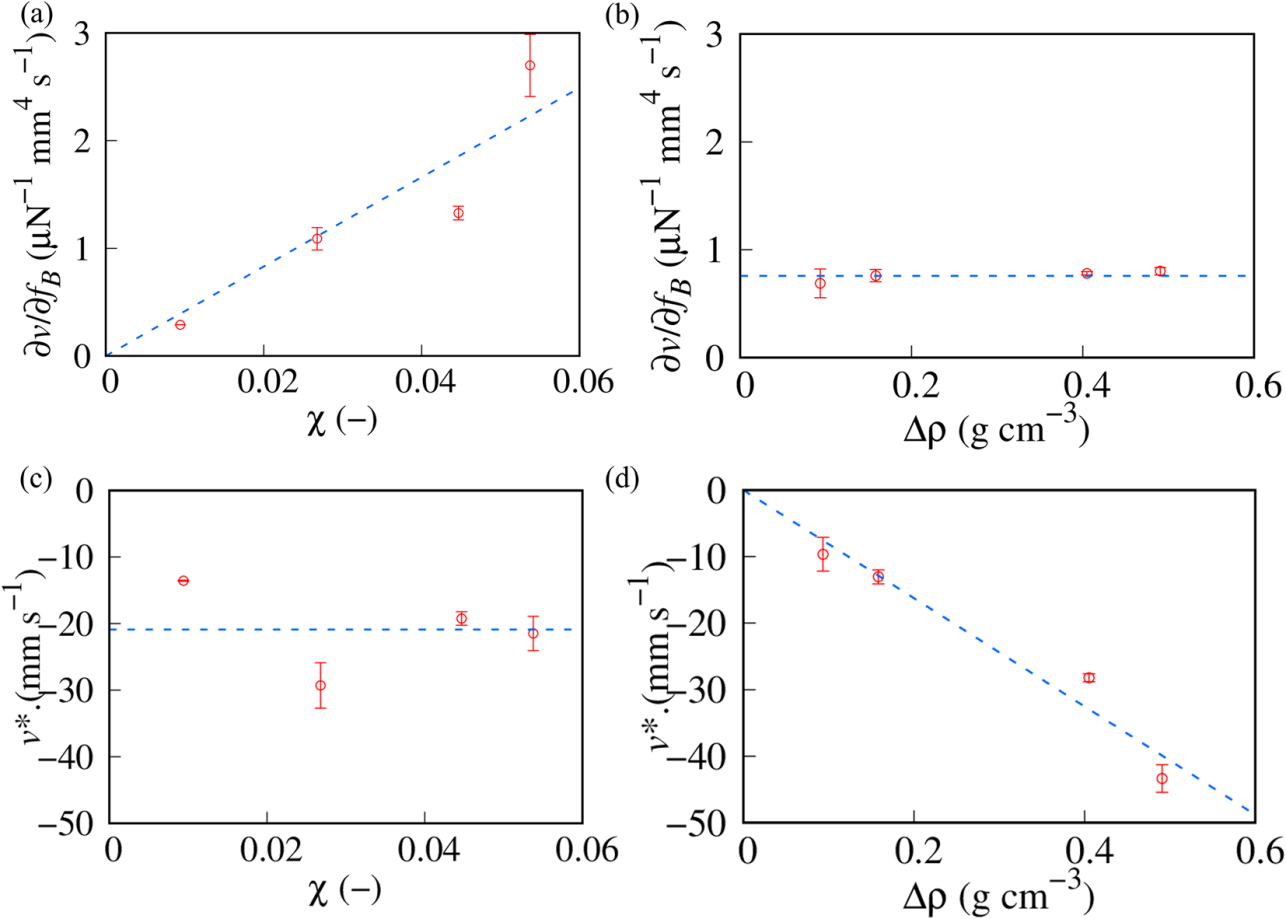
Quantitative analysis of transport dynamics of MPs. (**a**) χ dependence of ∂*v*/∂*f*_*B*_. The dashed line is the best fit obtained using the linear function (∂*v*/∂*f*_*B*_ = γ_1_ χ, where γ_1_ = 42 μN^−1^ mm^4^ s^−1^). (**b**) △ρ dependence of ∂*v*/∂*f*_*B*_. ∂*v*/∂*f*_*B*_ is constant (0.76 μN^−1^ mm^4^ s^−1^) independently of △ρ. (**c**) χ dependence of v*. v* is constant (− 21 mm s^−1^) independently of χ. (**d**) △ρ dependence of v*. The dashed line is the best fit obtained using the linear function (v* =  γ_2_△ρ, where γ_2_ = − 8.1 × 10^4^ mm^4^ s^−1^ g^−1^).

We compared the theoretical predictions with the experimental results. Figure 6a shows the χ dependence of ∂*v*/∂*f*_*B*_. ∂*v*/∂*f*_*B*_ was proportional to χ, while it was independent of △ρ, as shown in Fig. 6b. These results agree with the behavior predicted from Eq. (5) (∂*v*/∂*f*_*B*_ = *V* χχ_Fe3O4_/ξ). We defined the imaginary intercept −α*f*_B_* in Eq. (2) as v*. We summarized the χ and △ρ dependencies of v* in Fig. 6c and d, respectively. v* is proportional to △ρ independently of χ, which agrees with the theoretical prediction (v* = *V*△ρ/ξ). These agreements suggest that the transport dynamics of MPs must be determined by the balance between *F*_*B*_, *F*_*g*_, and *F*_vis_.

Let us discuss the important parameter *f*_B_*, which determines the resolution of the selective transport of MPs in microdroplets. Since *v* becomes positive with *f*_*B*_ > *f*_B_*, MPs are transported upward. In other words, *v* = 0 with *f*_*B*_ = *f*_B_*, which yields the following theoretical equation,

6 $$f_{B}^{*} = \frac{g}{{\chi_{{Fe_{3} O_{4} }} }}\frac{\Delta \rho }{\chi }.$$

Here, *f*_B_* depends on the density, and magnetic susceptibility. Since both *v* and *F*_vis_ were approximately equal to 0 in the vicinity of the threshold value (*f*_*B*_ ~ *f*_B_*), the relationship between magnetic force and gravity determined the direction of transport of MPs in microdroplets.

**Figure 7:**
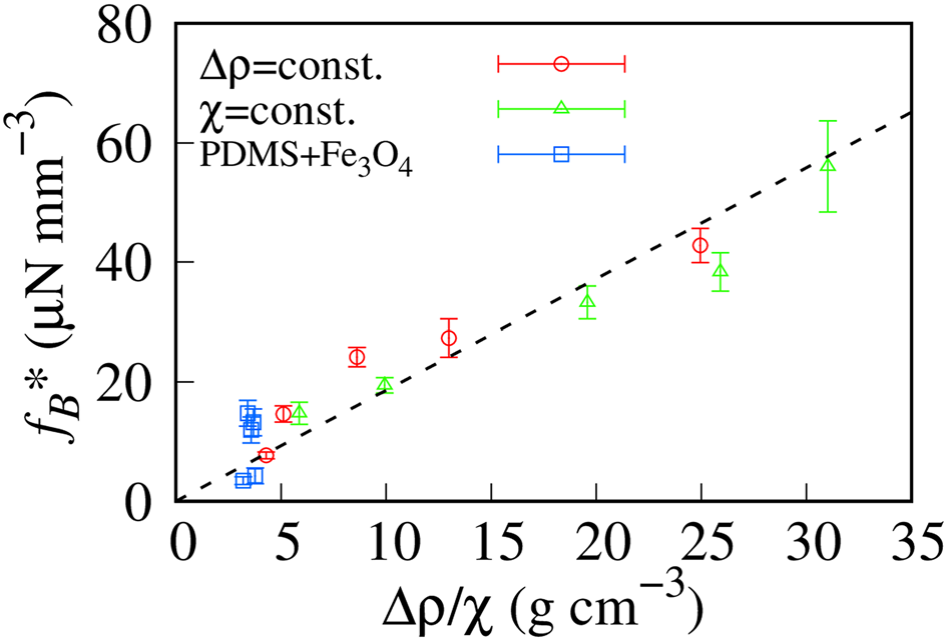
△ρ/χ dependence of *f*_B_*. Red and green markers indicate *f*_B_* with the condition of constant △ρ (Sample series 1) and constant χ (Sample series 2), respectively. Blue markers indicate *f*_B_* of the binary magnetized particles (MPs; Sample series 3). The dashed line is the best-fit curve obtained using a linear function (*f*_B_* =  δ△ρ/χ, where δ = 1.9 (±0.14) m s^−2^).

To compare the experimental results with the theoretical prediction, we summarized *f*_B_* obtained in Figs. 3b and 4b as a function of △ρ/χ. We superimposed *f*_B_* of the binary MPs composed of polydimethylsiloxane (PDMS) and Fe_3_O_4_ in Fig. 7. *f*_B_* distributes on a master curve despite the sample conditions, that is, constant △ρ or constant χ.

We fitted the experimental results with Eq. (6) and showed the best-fit curve as a dashed line in Fig. 7. The threshold curve agreed with the experimental results. Equation (6) suggests that the slope is *g*/χ_Fe3O4_. Substituting *g* = 9.8 m s^−2^, and χ_Fe3O4_ = 10–100^18,21^, we obtained *g*/χ_Fe3O4_ = 0.1–1 m s^−2^, which agrees with the experimental value obtained by fitting (~ 2 m s^−2^). Therefore, the threshold value of the magnetic transport of MPs in microdroplets depended primarily on the density and the magnetic susceptibility.

The resolution of *f*_B_* for the density (∂*f*_B_*/∂△ρ) was independent of △ρ. In contrast, the resolution for χ (∂*f*_B_*/∂χ) improved with decreasing χ; However, we required a high magnetic force to transport MPs. Thus, for MPs, such as magnetized cells, whose △ρ and χ are small, we may obtain high χ-resolution as well as a low magnetic force threshold.

We investigated the dynamics of single MP or sparse systems. Thus, the disturbance of a magnetic field generated from an electromagnet is small. Even in dense systems, if clusters of MPs can be regarded as one large particle, our findings should give us the criterion for selective manipulation. However, when we apply our method to dense and inhomogeneous systems, the local magnetic interaction between particles may be important, that is the disturbance of the magnetic field may be dominant. The effect of multibody interaction in dense systems on selective manipulation should be investigated in the future. 

## Methods

### Fabrication of the wetting pattern substrates

**Figure 8:**
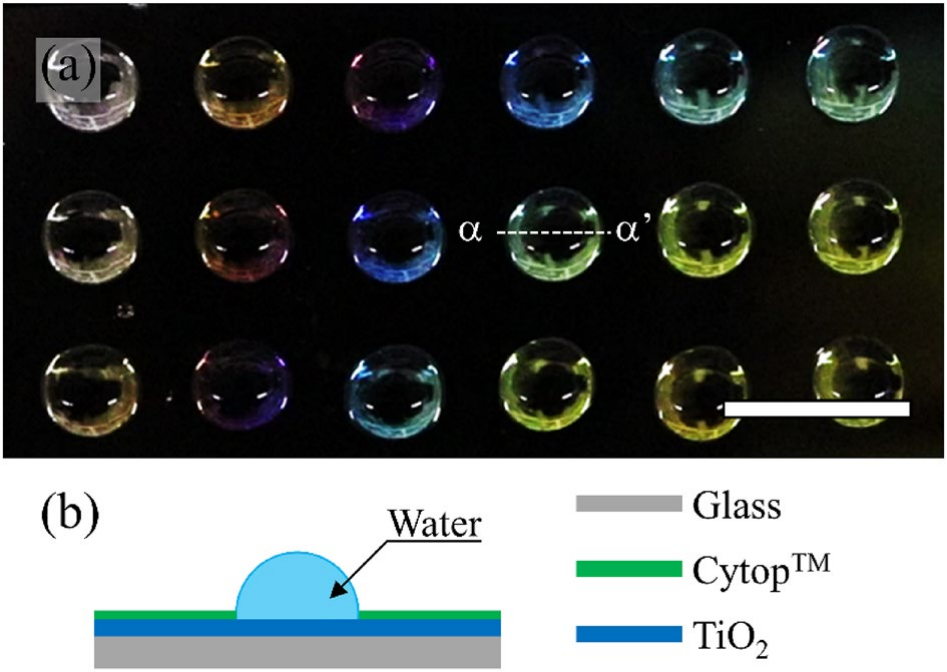
Microdroplets on a wetting pattern (WP) substrate. (**a**) Photograph of microdroplet array on a WP substrate. We formed 30 μL microdroplets on hydrophilic areas of a WP substrate. Scale bar indicates 10 mm. (**b**) Schematic α–α’ cross-section of a microdroplet on a WP substrate.

We fabricated the WP substrates to realize the VCC of microdroplets. The fabrication process of WP substrates has been reported in a previous work^5^. We used TiO_2_ and Cytop™ (AGC) as hydrophilic and hydrophobic materials, respectively. Figure 8a shows the picture of microdroplets on a WP substrate. We pipetted 30 μL of water onto individual hydrophilic areas to form hemispherical microdroplets. Figure 8 (**b**) shows schematic α–α’ cross-section of a microdroplet on a WP substrate. To obtain hemispherical microdroplets, we set the radii of hydrophilic areas to 24.4 mm.

### Fabrication of magnetized particles

We fabricated MPs composed of Fe_3_O_4_, CuO, and PDMS to control the magnetic susceptibility of the MPs independently of the density. We used Fe_3_O_4_ and CuO to modulate the magnetic susceptibility and density, respectively. Since the magnetic susceptibility of Fe_3_O_4_ is much higher than that of CuO and PDMS (△χ_Fe3O4_ >  > △ χ_CuO_ ~ △ χ_PDMS_), we can regard the magnetic susceptibility of MPs χ_MP_ as χ_Fe3O4_ *v*_Fe3O4_, where *v*_Fe3O4_ is the volume fraction of Fe_3_O_4_. We defined the relative magnetic susceptibility of MPs with Fe_3_O_4_ and χ_MP_ / χ_Fe3O4_ as χ. The densities of CuO, Fe_3_O_4_, water, and PDMS are 6.31 g cm^−3^, 5.17 g cm^−3^, 0.997 g cm^−3^, and 0.967 g cm^−3^, respectively.

We added Fe_3_O_4_ and CuO to PDMS with weight ratios *m*_Fe3O4_, *m*_CuO_, and *m*_PDMS_. We stirred the mixture in a condition mixer (AR-100, THINICY) for 120 s and obtained a uniform mixture composed of Fe_3_O_4_, CuO, and PDMS. We maintained the thickness of this mixture at ~ 1 mm at 80 ºC for 1 h and stiffened PDMS. Further, we fabricated cylindrical MPs with a diameter of ϕ = 1 mm by hollowing them out from the PDMS mixture sheet. Dipping MPs in 10 wt.% gelatin water solution, we hydrophilized the surface of MPs.

To investigate the effect of χ and ρ_MP_ individually, we fabricated the MPs following conditions: constant ρ_MP_ (Sample series 1, Table S1); constant χ (Sample series 2, Table S2). Binary MPs were composed of Fe_3_O_4_ and PDMS (Sample series 3, Table S3).

## Conclusion

In this study, we succeeded in selectively transporting MPs in microdroplets using a magnetic force. The application of a magnetic field to MPs introduced in microdroplets caused the transport of MPs against gravity. Further, we investigated transport dynamics focusing on χ and △ρ, which yields the threshold magnetic fields. Then, we analyzed the dynamic behavior of MPs with a force balance model. Our theoretical prediction agrees with experimental results. Thus, we concluded that the transport velocity was determined by a force balance between *F*_*B*_, *F*_*g*_, and *F*_vis_, while the threshold depended only on the ratio of the density to the magnetic susceptibility. If both density and magnetic susceptibility are known, the criterion of Eq. (6) can be applied to other systems, such as magnetized cells. In particular, our criterion can be applied to single or sparse systems such as magnetized spheroid in hanging droplets as it is. Therefore, our criterion for the selective transport of MPs in microdroplets is universal.

We demonstrated the separation of MPs into individual microdroplets by △ρ/χ. Combination of magnetic transport and DAST serves as a selective manipulation mechanism in biochemical applications. In this study, we showed the selective transport of three kinds of MPs. We may be able to expand the principle into a large-scale microdroplet array. For example, we can dispense magnetized cells cultured in microdroplets by their densities and magnetized susceptibility. Thus, the selective transport mechanism proposed in this paper may contribute to a high-throughput biochemical assay.

## Supplementary Information

Supplementary Information 1.

Supplementary Video 1.

Supplementary Video 2.

## Data Availability

The data that support the findings of this study are available from the corresponding author upon reasonable request.
