# Supplementary material for: Threshold magnetic field as a universal criterion for the selective transport of magnetized particles in microdroplets
Source: Sci Rep. 2023 Jun 9;13:9428. doi: 10.1038/s41598-023-36516-3 (PMC10256720; doi:10.1038/s41598-023-36516-3)
Supplement: Supplementary file 1 — Supplementary Information 1. [file 41598_2023_36516_MOESM1_ESM.docx]

Supplementary Information for

**Threshold magnetic field as a universal criterion for the selective transport of magnetized particles in microdroplets**

Shinji Bono, Satoshi Konishi

*Corresponding author. Email: [bono@fc.ritsumei.ac.jp](mailto:bono@fc.ritsumei.ac.jp)

**Supplementary Information 1: Calibration of the magnetic driving force**

We measure the magnetic field *B* generated by the electromagnet using a Tesla meter. The distance between the Tesla meter and the electromagnet is maintained in the range of 1.0 mm < *D* < 6.5 mm. Figure S1 shows *B* as a function of the applied current *I*. *B* is proportional to *I* and increases with decreasing *D*. We fitted the experimental results with a linear function of *I* and obtained the slope *B*/*I*.


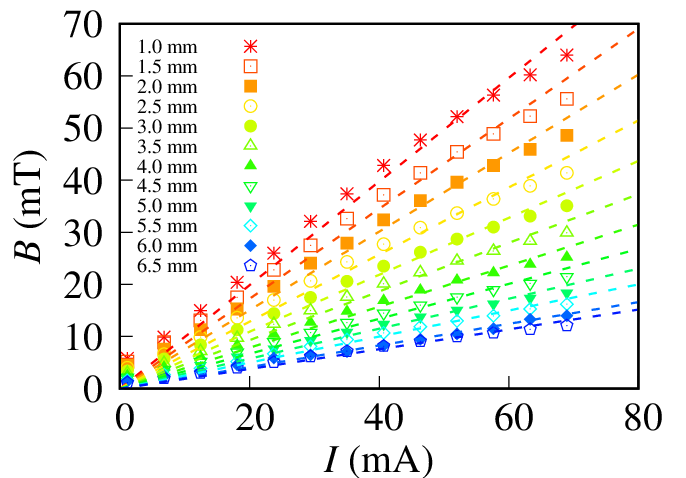


**Figure S1.** *I* dependence of *B*. The dashed lines are the best fits obtained using linear functions.

Figure S2 shows the *D* dependence of *B*/*I*. In our experiment, 3 mm < *D* < 5 mm, where *B*/*I* is proportional to *D* and the slope ∂*B*/∂*D* = − ∂*B*/∂*z* = − 0.13 T A^−1^ mm^−1^. Thus, the magnetic field defined in equation (1) is given as

$\text{f}_{\text{B}}\text{ }\text{=}\text{ }\frac{\text{1}}{\text{}_{\text{0}}}\text{B}\text{ }\frac{\text{}\text{B}}{\text{}\text{z}}\text{ }\text{=}\text{ }\text{0.10 }\left( \text{0.92+0.13}\text{z} \right)\text{I}^{\text{2}}\text{ }\text{}\text{N m}\text{m}^{\text{-3}}$ (S1)

We controlled *f_B_* by modulating *I*.


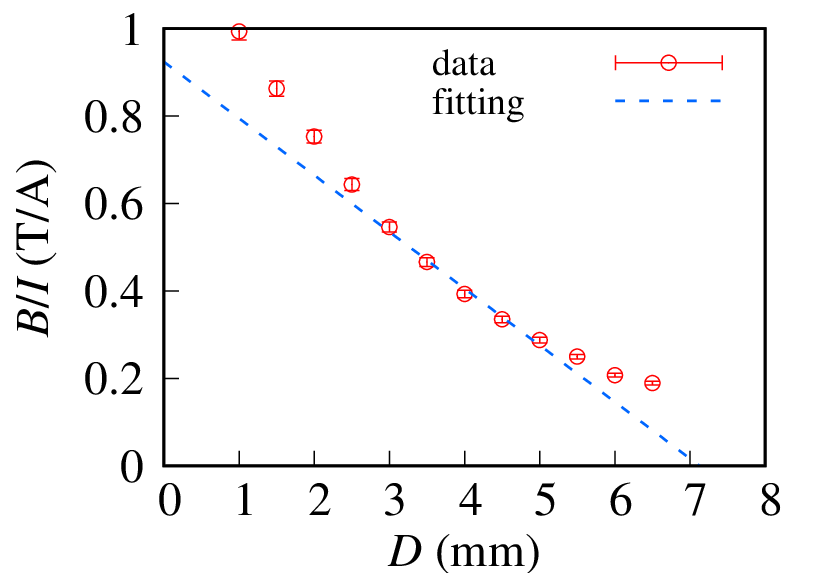


**Figure S2.** *D* dependence of *B*/*I*. We fitted *B*/*I* in the range of 3 mm < *D* < 5 mm with a linear function of *D* and shown as a dashed line. The slope and intercept are 0.13 T A^−1^ mm^−1^ and 0.92 T A^−1^, respectively.

**Supplementary Information 2: Magnetic susceptibility and density of magnetized particles**

**Table S1**: Physical properties of magnetized particles (MPs) composed of Fe_3_O_4_, CuO, and polydimethylsiloxane with constant density

| *m*_Fe3O4_ (wt.%) | *m*_CuO_ (wt.%) | *m*_PDMS_ (wt.%) | χ (-) | ρ (g cm^−3^) | Δρ (g cm^−3^) |
| --- | --- | --- | --- | --- | --- |
| 4.0 | 21.8 | 74.2 | 0.0094 | 1.23 | 0.23 |
| 7.5 | 18.1 | 74.4 | 0.0178 | 1.23 | 0.23 |
| 11.3 | 14.4 | 74.3 | 0.0268 | 1.23 | 0.23 |
| 18.8 | 7.0 | 74.2 | 0.0447 | 1.23 | 0.23 |
| 22.6 | 3.6 | 73.8 | 0.0538 | 1.23 | 0.23 |

**Table S2**: Physical properties of magnetized particles (MPs) composed of Fe_3_O_4_, CuO, and polydimethylsiloxane with constant magnetic susceptibility

| *m*_Fe3O4_ (wt.%) | *m*_CuO_ (wt.%) | *m*_PDMS_ (wt.%) | χ (-) | ρ (g cm^−3^) | Δρ (g cm^−3^) |
| --- | --- | --- | --- | --- | --- |
| 7.6 | 6.3 | 86.1 | 0.0159 | 1.09 | 0.093 |
| 7.1 | 12.6 | 80.3 | 0.0159 | 1.16 | 0.16 |
| 6.3 | 25.0 | 68.7 | 0.0159 | 1.31 | 0.31 |
| 5.8 | 31.2 | 63.0 | 0.0159 | 1.40 | 0.41 |
| 5.5 | 36.2 | 58.3 | 0.0159 | 1.49 | 0.49 |

**Table S3**: composed of Fe_3_O_4_, and polydimethylsiloxane

| *m*_Fe3O4_ (wt.%) | *m*_PDMS_ (wt.%) | χ (-) | ρ (g cm^−3^) | Δρ (g cm^−3^) |
| --- | --- | --- | --- | --- |
| 29.8 | 70.2 | 0.0734 | 12.7 | 0.28 |
| 26.1 | 73.9 | 0.0617 | 12.2 | 0.23 |
| 22.4 | 77.6 | 0.0510 | 1.18 | 0.18 |
| 18.6 | 81.4 | 0.0409 | 1.14 | 0.14 |
| 14.9 | 85.1 | 0.0318 | 1.10 | 0.10 |

**Supplementary Information 3: Description of supplementary movie**

**Supplementary movie 1: Manipulation of a magnetized particle in a coalescent droplet**

ρ_MP_ and χ of the magnetized particles (MPs) are 1.23 g cm^−3^ and 2.7 × 10^−2^, respectively. We applied *f_B_* = 5.9 μN mm^−3^. Scalebar indicates 3 mm.

**Supplementary movie 2: Selective manipulation of magnetized particles in microdroplets**

We summarize the physical properties of the particles in Table 1. We applied *f_B_*_H_ = 70 μN mm^−3^ while the top microdroplet contacted the left microdroplet. We applied *f_B_*_L_ = 10 μN mm^−3^ while the top microdroplet establishes contact with the center microdroplet. Scale bar indicates 5 mm.
